# Editorial: Role of non-coding RNAs in development and metastasis of solid tumours

**DOI:** 10.3389/fcell.2023.1281200

**Published:** 2023-09-25

**Authors:** Bappaditya Roy, Samikshan Dutta, Chandrama Mukherjee

**Affiliations:** ^1^ Department of Microbiology, The Ohio State University, Columbus, OH, United States; ^2^ Center for RNA Biology, The Ohio State University, Columbus, OH, United States; ^3^ Department of Biochemistry, University of Nebraska Medical Center, Omaha, NE, United States; ^4^ RNABio Lab, Institute of Health Sciences, Presidency University, Kolkata, West Bengal, India

**Keywords:** non-coding RNA, circular RNA (cirRNA), long non-coding RNA(lncRNA), solid tumour, metastasis

Non-coding RNAs (ncRNAs) including long non-coding RNAs (lncRNAs), micro RNAs (miRNAs), and circular RNAs (circRNAs) are emerging as key players in regulating tumorigenesis. Though these ncRNAs do not encode for functional proteins, they can act epigenetically, transcriptionally, post transcriptionally or at translational and post translational levels to alter gene expression. Accumulating evidence suggests that lncRNAs alter gene expression during the development and metastasis of solid tumors. Importantly, these non-coding RNAs are also used as biomarkers in different cancers. This special edition presents a wide Research Topic of reviews that summarize recent work in this area.

CircRNAs are generated by back splicing from covalently closed loops. The first circRNA was reported in virus (Kolakofsky, 1976; Sanger et al., 1976). Three years later, circRNAs were identified in the cytoplasm of several eukaryotic cells by electron microscopy by Hsu and Coca-Prados (1979). With the advancement of high throughput sequencing and bioinformatics, in-depth analysis, and characterization of circular RNAs have been emerging. CircRNAs usually are more stable than linear ones and can be translated into small peptides through cap-independent manner. The recently identified approximately 1,500 circular RNA in mouse or rat genomes unravel the possibility of the identification of peptides. In humans, the first circRNA was detected in Hela cells in 1979. After that, various functions of circular RNAs have been implicated in a number of diseases and disorders such as diabetes, neurological disorders, cardiovascular diseases, and cancer. In cancer, multiple studies suggest that peptides synthesized from the circRNA, play a critical role in proliferation, invasion and migration of various cancer cells. Recently, a circRNA produced by the SNF2 histone linker PHD RING helicase (SHPRH) gene was reported to be associated with various cancers including lung, bladder, liver, and colorectal cancers, and glioma. The review article by Xiong et al. summarizes the role of circ-SHPRE in regulating gene expression in tumorigenesis and as a prognostic biomarker for the early detection of various solid cancers.

LncRNAs regulate the expression of genes post-transcriptionally. LncRNAs can also modulate nuclear organization, mRNA processing, and protein activities and thus are known to play diverse roles in regulating different stages of cancer development. Macha and his group in this Research Topic describe how lncRNAs regulate signaling pathways and modify the expression of genes to alter the tumour microenvironment to promote metastasis. They have provided a detailed mechanistic analysis on the interaction of lcnRNAs with the cancer microenvironment. Baba et al. also highlight the possible usage of lncRNAs in treating cancer.

These review articles focus on various functions of lncRNAs in regulating gene expression. LncRNAs are often considered as promising reagents for future therapeutics too. LncRNAs function as signaling molecules to regulate transcriptional activity, act as decoys to titrate away proteins or other regulatory RNAs or guide the localization of ribonucleoprotein complexes and as scaffolds to assemble ribonucleoprotein complexes. LncRNA can also act as endogenous competitive RNAs of miRNAs that share sequence identity and similarity with mRNA and can competitively bind to miRNAs to exert their function. CASC11 belongs to this class of lncRNA and functions as a critical tumor promoter in various cancers. Ghafouri-Fard et al. focused on recent advancements in understanding the role of CASC11 in carcinogenesis and its potential clinical applications.

MINC is another lncRNA, identified recently from the TCGA database. It is associated with different types of cancer. It regulates cell cycle, proliferation and apoptosis. Multiple lines of evidence demonstrate elevated level of this lncRNA in various cancers including gallbladder, hepatocellular, colorectal, nasopharyngeal and non-small cell lung cancer, and in oral squamous cell carcinoma and glioma. Chao et al. outline the deficiencies in our present knowledge of the field and potential ways to explain the function of this RNA in the context of its oncogenic and other related properties. The study also provides evidence that MINC is often considered a prognostic biomarker in various cancers.

In summary, this Research Topic focusses on the dynamic functions of ncRNAs in regulating development of metastasis of solid tumors and also highlights the potential therapeutic use of ncRNAs.
